# Experimental Pain Phenotypes in Older Adults with Knee Osteoarthritis: A Neural Network-Based Clustering Approach

**DOI:** 10.1093/geroni/igaf122.3373

**Published:** 2025-12-31

**Authors:** Chiyoung Lee, Juyoung Park, Heewon Kim, C Kent Kwoh, Xiaoxiao Sun, Chen X Chen, Hyochol Ahn

**Affiliations:** University of Arizona, Tucson, Arizona, United States; University of Arizona, Tucson, Arizona, United States; Soongsil University, Seoul, Republic of Korea; University of Arizona, Tucson, Arizona, United States; University of Arizona, Tucson, Arizona, United States; University of Arizona, Tucson, Arizona, United States; University of Arizona, Tucson, Arizona, United States

## Abstract

Research has emphasized the “phenotyping” of knee osteoarthritis (KOA) pain as a priority to effectively target therapies to individual patients. Following this initiative, this study aimed to characterize pain phenotypes based on experimental pain responses in older adults with symptomatic KOA. We utilized baseline data from a clinical trial that combined non-invasive neuromodulation and meditation in its multimodal approach (N = 200). Participants completed demographic and clinical questionnaires, followed by a multimodal quantitative sensory testing (QST) battery. For phenotyping, we implemented a two-layer neural network-based k-means algorithm. Four phenotypes emerged, showing significant differences across QST measures (p < 0.001) and were characterized as: (1) high pressure pain thresholds and high conditioned pain modulation (indicating low sensitivity to pain and efficient descending inhibition); (2) average pain responses across most QST modalities; (3) low pressure pain thresholds, high punctate mechanical pain, enhanced temporal summation of pain, and low conditioned pain modulation (indicating full manifestation of peripheral, spreading, and central sensitization along with deficient descending inhibition); and (4) low heat pain thresholds, low heat pain tolerance, and high cold pain (indicating high sensitivity to thermal stimuli). Phenotypes differed by gender, marital status, pain severity (measured by the numeric rating scale), and KOA-related symptoms (measured by the Western Ontario and McMaster Universities Osteoarthritis Index) (p < 0.05). Our findings suggest that older adults with symptomatic KOA should be treated according to their phenotypes. Additionally, the identified phenotypes may be useful for selecting and stratifying patients in clinical trials evaluating analgesic compounds and non-pharmacological interventions.

